# Fungemia due to *Aureobasidiumpullulans*

**DOI:** 10.1016/j.mmcr.2022.06.004

**Published:** 2022-07-05

**Authors:** Jorge Verdecia, Christopher A. Jankowski, Meredith L. Reynolds, Yvette McCarter, Malleswari Ravi

**Affiliations:** a Department of Medicine, Division of Infectious Diseases, University of Florida, Jacksonville, USA; b Department of Pharmacy, University of Florida, Jacksonville, USA; c Department of Microbiology, University of Florida, Jacksonville, USA; d Department of Pathology, University of Florida, Jacksonville, USA

**Keywords:** Aureobasidium spp, Fungemia, Catheter associated infection, Posaconazole, Caspofungin

## Abstract

*Aureobasidium pullulans* is a yeast-like dematiaceous fungus ubiquitous in nature. It is a rare cause of skin and soft tissue infection, peritonitis, and catheter-related fungemia in certain human hosts. We report a case of recurrent *A. pullulans* catheter-related fungemia that was successfully treated with caspofungin, posaconazole, and catheter removal.

## 1 Introduction

*Aureobasidium pullulans* is a saprophytic yeast-like dematiaceous fungus with melanotic pigments in its cell wall [[1], [2], [3], [4],^6^]. It is ubiquitously found in soil, plants, and moist surfaces such as glass. It has also been reported to colonize human skin [[1], [2], [3], [4],^7^]. It is a rare cause of skin infections, meningitis, splenic abscesses, peritonitis, and fungemia often associated with indwelling intravenous catheters [1,2,[4], [5], [6]]. This is a case of recurrent *A. pullulans* fungemia in a patient with a chronic indwelling central line that was successfully treated with caspofungin and posaconazole in addition to catheter removal. This case is important as there is limited literature on this pathogen and appropriate therapy.

## 2 Case

A 60-year-old male with a past medical history of heart failure with an ejection fraction 25–30% NYHA II on home milrinone through a peripherally inserted central catheter (PICC), biventricular-ICD in-situ, uncontrolled type 2 diabetes, and history of multiple episodes of bacteremia that presented to the emergency department (ED) due to one day of fever noted at home. In the ED, fever peaked at 103.2 °F (39.5 °C) and improved with acetaminophen. Physical exam was remarkable for acute distress and tachycardia but no tenderness, erythema, or purulence at the catheter site. Pertinent initial laboratory findings were a white blood cell count of 9840 per microliter, with normal renal and liver function. He was admitted to the medical floor, blood cultures were collected and empiric vancomycin and cefepime were begun on day 0. Two sets of blood cultures from the PICC line and peripheral venipuncture were positive at days 3 and 4 of incubation, respectively, with yeast suggestive of *Candida* spp. Per hospital Infectious disease protocol, he was started on intravenous caspofungin 50 mg/d on day three. Broad-spectrum antibiotics were discontinued. Repeat blood cultures were found to be negative and the infected PICC line was removed on day 4 and replaced with a new PICC line. Infectious disease team was consulted on day 7 when the organism was identified as *A. pullulans*. Since the patient remained afebrile on day 6, and had negative blood cultures, it was decided to continue caspofungin 50 mg/d for 14 days. Susceptibilities were requested but were not available until 3 weeks later (Table 1). The PICC line tip was not sent for culture. Upon questioning, the patient reported being active at home by doing all his chores without assistance, including yard work.

**Table 1:** Antifungal susceptibility profiles of *A. pullulans* identified from case patient compared to published literature. MIC ug/ml.

	1st episode Isolate, (n = 1), MIC	2nd episode Isolate, (n = 1), MIC	Mittal et al.^2^ Isolate (n = 1), MIC	Najafzadeh et al.^7^ Total isolates (n = 104) MIC _90_ (Range)
Amphotericin B	–	0.5	0.12	1 (≤0.016–16)
Caspofungin	>8	>8	<0.015	4 (0.063–8)
Fluconazole	16	32	8	≥64 (4-≥ 64)
Posaconazole	0.06	0.25	0.015	0.5 (≤0.016–4)
Itraconazole	–	–	–	0.5 (≤0.016–16)
Isavuconazole	–	4	–	4 (0.016–16)
Voriconazole	0.06	0.125	0.12	2 (≤0.016–16)
Terbinafine	–	1	–	–

A month later, he was admitted for malaise and found to have coagulase-negative *Staphylococcus* bacteremia, for which a central catheter was exchanged. After that, he was admitted for multiple episodes of heart failure. Fungal and routine blood cultures obtained during those admissions were negative. However, six months after the initial fungemia presentation, the patient was admitted with fever, chills, and generalized fatigue. Gram stains of the two blood culture sets were reported as yeast suggestive of *Candida* spp. Given prior episode of fungemia, the microbiology laboratory suspected *Aureobasidium* spp., and the identification was confirmed as *A. pullulans* var. *pullulans* using MALDI-TOFF. The specimen was sent to a reference laboratory for susceptibility testing (Table 1). Initially, caspofungin 50mg/d was started but was rapidly transitioned to oral posaconazole 300 mg/d based on previous susceptibility testing results. Unfortunately, the patient deteriorated due to worsening heart failure and expired four weeks after admission.

## 3 Discussion

**Fig. 1:**
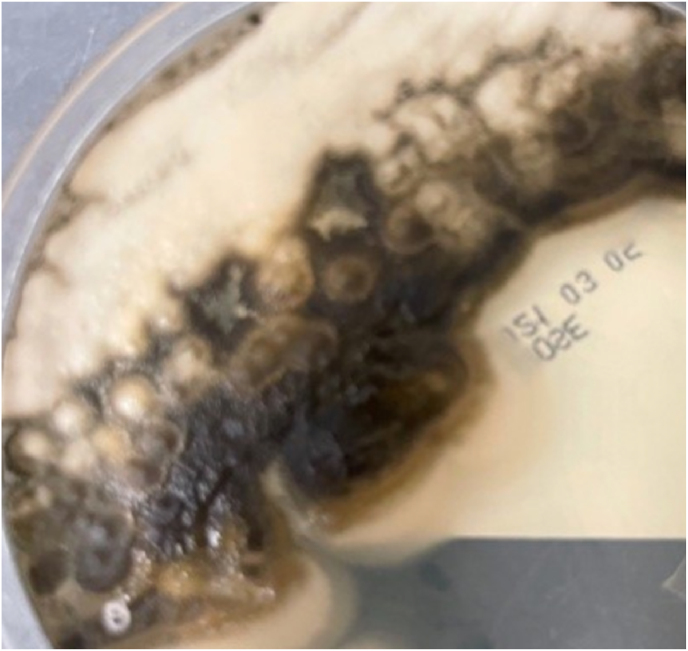
Aureobasidium spp growing on Sabouraud dextrose agar.

**Fig. 2:**
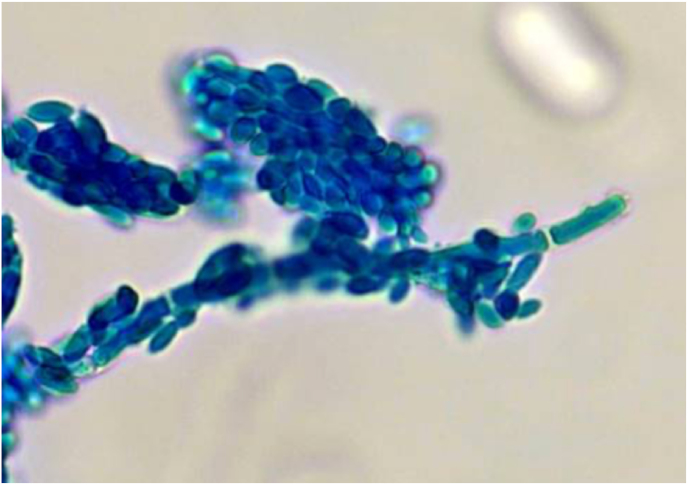
A lactophenol cotton blue preparation of colonies initially showing hyaline, septate, and thin-walled hyphae. (For interpretation of the references to colour in this figure legend, the reader is referred to the Web version of this article.)

**Fig. 3:**
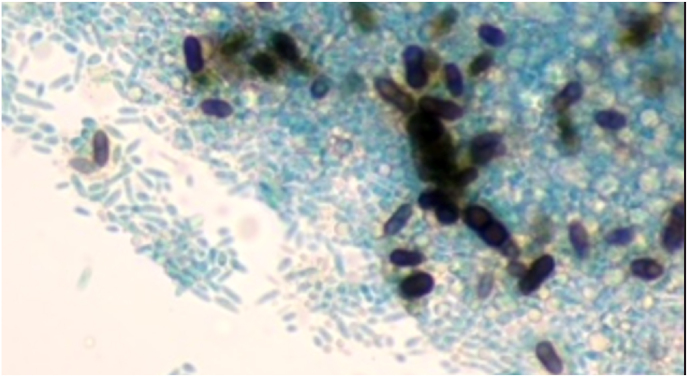
Mature colonies demonstrate thick-walled arthroconidia, which darken due to melanin on lactophenol colon blue preparation. (For interpretation of the references to colour in this figure legend, the reader is referred to the Web version of this article.)

The genus *Aureobasidium* contains a single species and several varieties containing differing amounts of melanin and having various salt and temperature tolerances [5]. Diagnosis of this organism can be difficult as it changes morphology depending on the age of the colony, temperature, carbon source, light, and substrate [2,3]. It grows on Sabouraud dextrose agar (Fig. 1) [3,7]. It has been reported in the literature that on initial gram stain, it tends to look like budding yeast because conidia form directly as buds from the hyphal wall [[1], [2], [3]]. Colonies initially show hyaline, septate, and thin-walled hyphae (Fig. 2) [1,3]. As the culture matures, in about 7 days, it forms thick-walled arthroconidia, which darkens due to melanin, as seen in Fig. 3 [5,7]. Melanin is thought to be a virulence factor, but the mechanism of production is unclear, see Fig. 1 A and B [5]. Melanin is known to protect the fungus against agents such as free radicals, toxic metals, and ionizing radiation [5]. The organism has traditionally been identified using microscopic morphology. Molecular methods have been used in some reports for organism identification [2,[4], [5], [6], [7]]. Matrix-Assisted Laser Desorption Ionization Time of Flight mass spectrometry [MALDI-TOF MS] can also identify this organism [3]. In our case, MALDI-TOF MS, but not molecular, was available to confirm identification of both specimens as *A. pullulans* var. *pullulans*.

There was no systematic data on *A. pullulans* antifungal susceptibility until 2014 when a comprehensive study was published that identified the organisms to the variety level: *A.pullulans var. pullulans* and *A. pullulans* var. *melanigenum* [7]. Based on the data collected by Najafzadeh et al., most human diseases have been caused by A. pullulans var. melanigenum rather than A. pullulans var. pullulans [7]. They performed in-vitro antifungal susceptibility testing on 104 environmental and clinical isolates, of which 38 were clinical isolates from the United States. Isolates were confirmed using the internal transcribed spacer region and D1/D2 region of rRNA [7]. Their isolates’ minimum inhibitory concentrations (MIC) were similar to the data published in the European Society of Clinical Microbiology and Infectious Diseases (ESCMID) guidelines published in April 2014 [8]; although, this data originated in a study with only 2 tested *Aureobasidium* spp. that were grouped together with other black molds [9].

The antifungal susceptibilities of the clinical isolates from our case patient were compared to previous studies in Table 1 [2,7]. In the Najafzadeh study the MIC_90_ for caspofungin was 8 μg/ml for *A. pullulans* var. *pullulans* and 2 μg/ml for *A. pullulans* var. *melanigenum*, respectively. Our patient's isolates had reduced caspofungin activity with MICs identified to be ≥ 8 μg/ml. In contrast, Mittal et al. reported a caspofungin MIC <0.015 μg/ml. In the Najafzadeh et al. study fluconazole demonstrated a high MIC_90_ of ≥64 μg/ml. However, our isolates had fluconazole MICs similar to Mittal et al. Currently, there are no CLSI interpretive criteria for *A. pullulans*. Nevertheless, amphotericin, itraconazole, and posaconazole had the greatest activity against *Aureobasidium* spp. than other antifungal agents based on the published literature [7]. In our patient amphotericin was not used in the first fungaemic episode. The reference laboratory did not perform amphotericin susceptibilities on this isolate due to a lab error. In addition, our patient was at greater risk for renal toxicity due to his comorbidities, and he clinically improved with caspofungin and catheter removal. However, there are case reports showing resolution of infection with catheter removal without the need for antifungal therapy [4].

From 1986 to 2021, there have been 26 cases of *A. pullulans* infection reported in the published literature, including this case [6]. Chamroensakchai et al. described 25 patients with an underlying condition, therapy, catheter, site of infection, and outcome [6]. Mortality was assessed by dividing the deceased patients [total of 6] and cases, resulting in all-cause mortality of 20%, which is significant. We believe our patient expired due to heart failure complications unrelated to the second fungaemic episode. Nonetheless, it is possible that the fungemia exacerbated the heart failure.

Due to poor hygiene, his PICC line was likely infected during his house and yard work. The organism was not thought to be a skin colonizer on either episode as the patient presented with signs of infection.

The patient was seen in follow-up in the infectious disease clinic about 2 weeks after hospital discharge from the first fungaemic episode, and he remained asymptomatic. He continued to receive milrinone via a central venous catheter while waiting for a heart transplant or left ventricular assist device. All repeat routine blood and fungal blood cultures remained negative at the first follow-up.

We believe this case is significant as systemic infection with *A. pullulans* is rare, and only about 26 cases have been described since the 1980s. Also, this would represent a third case that was successfully treated with an echinocandin in addition to catheter removal.

In summary, *A. pullulans* appears to be an emerging pathogen within the last four decades. Factors that can influence the rise of this organism include increased use of central lines, especially in immunocompromised patients, and improved lab identification techniques. New identification techniques such as molecular and MALDI-TOF MS allow for faster and more accurate identification but are not readily available in most institutions. There is some guidance from the European Society of Clinical Microbiology and Infectious Diseases recommending the use of amphotericin B. However, the guidelines do not offer insight into alternative drug choices or the duration of treatment due to a lack of evidence. Yeast in blood cultures in appropriate hosts should raise suspicion for fungal species other than *Candida* as candidemia first-line treatment is caspofungin which may not be suitable for an organism like Aureobasidium.

## Declaration of competing interest

There are none.
